# A Clinical Treatise on the Endemic Fevers of the West Indies

**Published:** 1837-04-01

**Authors:** 


### A Clinical Treatise on the Endemic Fevers of the West Indies, intended as a Guide for the Young Practitioner in those Countries. By *W. J. Evans*, Esq. M.R.C.S. Octavo, pp. 309. Churchill, London, 1837.

The object of our author is very laudable—though, perhaps, of difficult attainment—"to exhibit the cases (of endemic fever) as they have occurred to me in all their varieties—to detail faithfully their symptoms—to expose the appearances that the different organs presented after death—and to explain, as far as I am able, the physiological operation of the producing causes." This is indeed an arduous task! The author considers that all the diseases of the West Indies, included under the term "*fever*," may be traced to one of three sources—malaria, solar heat, and contagion. The topography of St. Lucia is then introduced, to exemplify the two first causes—the third, contagion, he has never witnessed.

In the third chapter is given a succinct account of all that is known respecting that mysterious agent—malaria—an agent hitherto inscrutable in its nature, though but too well recognized in its deleterious effects on the human frame. Our author divides the physiological effects of malaria into two heads—its agency on the acclimated and unacclimated population. We here introduce a short extract from the first division.

"The inhabitants of marshy countries, where the temperature is moderate, present the following appearances:—The skin is pale, livid, or yellow, the eyes dull and heavy, the movements of the body slow and languid, the eyelids and face puffy and somewhat swollen, or shrunk and wrinkled at an age when they ought not to be; the abdomen large, the extremities small, shrunk, and wanting consistence in the muscles; the sensations both of pleasure and pain seem deadened; common events produce little effect, exciting neither joy nor sorrow. They present the lymphatic, or, as it was formerly called, the leuco-phlegmatic temperament, in its greatest development. Cadaveric inspection shews us chronic engorgements or morbid changes of structure in some or all of the viscera. Acute inflammations are rare, but, living in an unwholesome atmosphere, they never enjoy health; they are always valetudinarians. How can it be otherwise, attacked as they so frequently are by intermittent or remittent fevers, which, though so slight as to be treated by the unfortunate beings who have served a long apprenticeship to suffering, with indifference, nevertheless ultimately produce premature old age and death? They suffer from neuralgic affections; sciatica and tic douloureux are common; their blood is thin and watery, giving rise to ulcers of the legs and scurvy.

As we approach the tropics, though the lymphatic temperament still obtains, we find it combined with a greater excitability of the nervous system; and within these regions are added irritability of the skin and of the abdominal viscera. There is a strange mixture of indolence and love of excitement: a wish to be roused from the listlessness of every-day life, but requiring a powerful stimulus to effect it. Slight causes produce affections of the nervous system which terminate in death. Colic, followed by paralysis of some part of the body, is common: and hysteria and other diseases of this class are frequently combined with the ordinary disorders of the country. We can account for this only by admitting a peculiar modification of the ordinary morbific agents, or a peculiar idiosyncrasy in the people." 17.

The following passage shews that our author is an attentive observer:—

"An European, or a native, after a long residence in a temperate and healthy climate, arriving in St. Lucia, complains of a feeling of weight in the atmosphere—a something which resists the wished-for exertion or exercise. Both his mind and body are oppressed; his intellect is clouded; his spirits are low and desponding, and all pre-existing love of enterprize vanishes. If his residence be protracted he has slight febrile movements, which come on regularly or irregularly, not sufficiently severe to prevent him pursuing his usual avocations, but which nevertheless are sufficient to induce him to throw himself upon a sofa, and require a powerful effort of resolution to combat. In this manner his body may gradually accommodate itself to the climate, but he may consider himself fortunate if he escape so easily; in general, particularly if he be guilty of any imprudences, he feels restless at night, and can only sleep during the cool of the morning; he feels out of sorts; has pains in the back and extremities, as if from fatigue; he complains of headache, sickness, and nausea; and if these symptoms are not attended to immediately, suffers what is vulgarly called an attack of seasoning fever.

When the constitution is affected by malaria in a still more powerful manner, the effects are often immediate, and I will describe them as they occurred to myself. I had occasion to visit an estate in the neighbourhood of Castries about midnight, and my road obliged me to pass the swamp which lies to the north of the town. There was a lovely moonlight, the sky was unclouded, and a heavy dew was falling. On approaching the swamp I was sensible of an extremely disagreeable odour, arising from its emanations, and from the dank and foul vegetation on its surface and in its neighbourhood. I then perceived a peculiarly unpleasant, but indescribable taste in my mouth and pharynx, which produced slight vertigo, nausea, and even efforts to vomit. On my arrival at the estate, which was shortly afterwards, I took a glass of hot punch, with the hope that it would allay the nausea; I was, however, mistaken, for it was immediately rejected, and an ague came on, which obliged me to go to bed, where I remained until the afternoon of the following day. After a profuse perspiration I was enabled to ride to town, though still very unwell. At night the symptoms returned with such severity, as to require a bleeding from the arm and leeches to the epigastrium; by these means the paroxysm subsided eighteen hours after its commencement, and a return was prevented by the free use of the sulphate of quinine." 20.

The terrific effects of concentrated malaria will be seen in this extract:—

"About eight o'clock in the evening two boatmen, after finishing their day's work, before returning home, were occupied in hauling their canoe high up on the beach, close to the most dangerous part of the swamp mentioned above, when they perceived immediately to windward a small cloud of vapour gradually approaching them, and in a short time they were enveloped in it. One of them fell down, apparently in a state of asphyxia, and the other was so affected as to be unable to render him any assistance. The vapour passed away quickly, and the one who had been the least incommoded recovered sufficiently to look after his companion, whom he found lying in the mud, apparently insensible. He continued in this state only a short time, and gradually became sufficiently well to be led home. In the course of the night Dr. Chevalier was called to see him, and found him suffering from an intense ague. The surface of the body was cold, the countenance expressed great anxiety, the pulse was small and scarcely perceptible, the patient was insensible to surrounding objects, and in a state of coma, only interrupted by severe convulsions. The cold stage continued altogether about three hours, and as reaction took place the convulsions subsided; but the coma continued, and alternated with delirium. There was occasional vomiting of mucosities with great effort and apparent pain. Pressure on the epigastrium made the patient wince and contort his countenance. About eighteen hours from the commencement there was remission, or rather a slight mitigation of symptoms, with a partial return of consciousness; it continued only a very short time, and was followed by another paroxysm, equal in violence to the last, except that the cold stage was scarcely perceptible. The patient died about forty hours from the period of exposure. The body was examined whilst warm; the blood was found fluid, and a small quantity of troubled serum was effused between the arachnoid and pia mater on the surface; the lungs were somewhat engorged, and the stomach intensely inflamed, containing about two or three ounces of blood in its cavity.

The other man never suffered further inconvenience; he said that the vapour had no perceptible smell; that it was warm and moist, like steam, stopped the respiration for a moment, and produced a sense of faintness and trembling of the whole body." 22.

Many other curious and fearful illustrations of malaria in its more deadly states of concentration are given by our author.

Our author's chapter on the effects of solar heat on the animal economy is a good digest of what is known on the subject, with some original observations. The disciples of Broussais consider insolation as almost the sole primary cause of tropical fevers—the excitement of heat on the surface being propagated to the stomach and bowels, and there kindling up fever. Mr. Evans allows this agent a share in the production of fever, but wisely attributes much to malaria.

"The effects of an elevated temperature on the human body are the following: —1st. An accelerated circulation, giving rise to a florid appearance of the blood from the short period of its sojourn in the capillaries; in other respects the properties of this fluid do not appear to be altered. 2nd. An irritation and inflammation of the skin, and a consequent exanthema known by the name of prickly heat, or the lichen tropicus of Dr. James Johnson. 3rd. Excitement of the chylopoietic viscera, particularly the mucous membrane of the stomach and intestinal canal, and the liver. 4th. Irritability of the nervous system."

Again:—

"When malaria is the exciting cause of disease, solar heat is combined with it, and the united effects are more virulent in consequence; though there may be intense inflammation of one or more organs, the general symptoms will not be those of inflammatory fever, properly so called. The blood will probably be found dark, fluid, and uncoagulable.

On the other hand, when inflammation of an organ, as the meninges, arises from insolation, or from some other cause independent of malaria, the symptoms will be those of a local inflammation, more or less modified, however, by this agent, in proportion to the violence of the inflammatory action on the one hand, and on the other, to the extent to which the system may be under its influence. Thus we may have a continued, remittent, or intermittent fever, having its point of departure in a local inflammation.

Not only does solar heat enter into the formation of disease by predisposing the body to receive it, or to be acted upon by morbific agents, but to the *existence* of the most common amongst these, malaria, it is absolutely essential; and, *cæteris paribus*, the one is in direct ratio with the other. In many countries in the north of Europe, the diseases which arise from malaria are almost unknown, and yet in them the marshes are so numerous and so extensive, as to render them perfectly uninhabitable were they exposed to the temperature of the tropics." 46.

Several cases are detailed in illustration; and amongst these are some of intermittent inflammations, which are curious. We shall extract a case of "intermittent pleuropneumony," as a sample.

"Lucille, a girl of colour, aged 23, has been unwell for two or three days with what she calls a cold, but has not been confined to the house until yesterday, when she was suddenly attacked with a severe pain in her side, cough, and fever, about nine o'clock in the morning. The symptoms ceased toward evening, and she remained tolerably well until this morning, August 21, 1833, when they again returned. At noon I found her as follows: Pain over the sixth and seventh ribs on the right side, increased on inspiration: dry cough, crepitating *râle* over the seat of the pain; no uneasiness of the epigastrium; bowels regular; skin dry and warm; tongue natural. She had no cold stage, nor was the attack preceded by a sense of coldness. *Diet—demulcent drink; castor oil.* Evening; the patient is perspiring; expectoration on coughing; the pain has subsided; the *râle* is mucous. 22nd. The patient has had a return of the paroxysm; the pain in the side is greater than yesterday; several fits of coughing, with expectoration of small quantities of very viscid mucus; *râle crepitant;* pulse ninety-six. Evening; perspiration, and mitigation of symptoms; expectoration free; the sputa white and frothy; there is still some pain on inspiration; the pulse is eighty-four; bowels open. 22. The paroxysm commenced two hours before its usual period; I saw the patient at noon; she is much worse than she has yet been; the sound on percussion over the seat of the disease is somewhat dull; breathing very painful. Evening; the disease is advancing; there is no appearance of intermission; sound on percussion dull; centre of the diseased part does not appear permeable to air: at least the crepitating *râle* in its neighbourhood hides the sound, if any exists; sputa viscid, adherent, and of a rusty colour, V.S. ℥xvi.; hirudines xx. lateri; decoction of marsh-mallow, in which antim. tart. gr. vi. are dissolved for drink. 24. The patient is better: to continue. Evening; amelioration very great; great prostration from the effects of the antimony, of which the patient has taken nearly eighteen grains. 25. Expectoration free; *râle* mucous; pain nearly gone; pulse seventy, small. To take the medicine only now and then. 26. Convalescent." 55.

We are inclined to demur to this example of "intermittent inflammation." No doubt the thoracic *inflammation* was aggravated whenever the intermittent *fever* came on; but we do not believe that the actual inflammation ever intermitted in its course, till convalescence was established. If it had been a real intermittent inflammation, why was not bark or arsenic employed?

A case of intermittent rheumatism is given at page 55, which, in our minds, is at least equivocal. We know that acute rheumatism will be one day in one joint, and another in another; but this is metastasis rather than intermittence.

Case IX. of "*intermittent peritonitis,*" is certainly more specious than any of the others; but still, a perusal of it inclines us to think that the inflammation went on steadily, from beginning to end of the case, and that the *painful phenomena* only, were suspended occasionally when the *fever* itself intermitted. The case, however, we shall present to our readers for their attentive consideration.

"A. V., aged 25, of a nervo-sanguineous temperament, superficial veins very much developed; native of the West Indies, but has resided until within a few months in France. Plunged into cold water when the body was very much heated, and shortly after eating a copious breakfast; immediately felt a general uneasiness, and vomited his breakfast; he was then seized with colicky pains of the belly, which continued nearly the whole day; in the night these pains became continued, and fever was not long in making its appearance. The following morning I saw him: the patient complains of considerable pain throughout the whole of the abdomen, nausea, violent efforts to vomit, skin hot and dry; no evacuation of the bowels for two days; pulse full, and 100 strokes in a minute; no head-ache; urine high-coloured, and small in quantity; the abdomen is very slightly distended, and extremely painful on pressure. V. S. ℥xvi. statim, castor oil; hot fomentations to the abdomen. Evening; the patient seems nearly well; the skin is moist, the pulse quiet, and the pain in the abdomen gone; he can bear pressure, though a little sensibility still remains; the bowels have not been opened; the castor oil was returned.—To take calomel, gr. x. to-night, and a black draught early in the morning. 20th. Noon; I have again been sent for, as all the symptoms returned at ten o'clock this morning, after an intermission of twelve hours; skin intensely hot and dry; pulse 108, full and hard; abdomen extremely painful; the weight of the sheet is almost insupportable; vomiting of bilious matter; great thirst. Bowels have been freely opened. I must confess that I feel a great deal of astonishment, as I looked upon the disease as completely vanquished last night, and little dreamed of its return in so severe a form. I considered it a case of peritoneal inflammation, from hyperhemia of this membrane, consequent upon the sudden constriction of the cutaneous capillaries produced by the cold bath. The blood taken away yesterday was very sizy. Bleeding repeated; hot fomentations; barley-water. Evening, blood cupped, buffy. As the patient could not bear the weight of the flannels, his friends put him into a warm-bath, which procured some relief; but he is still much in the same state as this morning, and there is now evidently a gastrite superadded; the tongue is red at tip and edges; great thirst; vomiting of bilious matters and mucosities, with effort; frontal head-ache, and heat and dryness of the skin. Hirudines xxx. abdom. During the application of the leeches the patient suddenly began to feel relief: the skin became moist, and the symptoms subsided rapidly. To take three grains of quinine every hour. 20th. The patient has passed an excellent night. There has been another intermission, but he has not taken any of the quinine; unfortunately his friends have thought proper to judge for themselves, and I was told that "*le sang est trop échauffé;*" he is now just like a person convalescent. There is a complete apyrexia, pulse soft, 80, skin moist; has perspired freely, and had his linen changed in consequence; but the abdomen is somewhat distended and sensible on pressure, though not much so, nor more than might be expected from the symptoms of yesterday. To take ten grains of quinine immediately, and to have them repeated in half an hour. Noon; all the symptoms returned half an hour after I saw him, and are now as violent as yesterday. To have a warm-bath immediately. The disease being decidedly intermittent, I think it injudicious to repeat the bleeding, as we may expect another intermission to-night. Nine o'clock P. M. I have been obliged to repeat the bleeding. The symptoms are more violent than ever; both the peritonitis and gastritis are most acute. Rep. hirudines. 21st. I have been up all night with this poor person. He has been again bled, and placed in a warm-bath until fainting was produced, but the symptoms have made an awful advance. Has had one stool, about eight hours since, from the use of a syringe. From midnight until about two o'clock in the morning there was a partial calm, and quinine was administered in a clyster, but was soon returned. Since this period the symptoms have gradually but rapidly increased, and at present, one o'clock, P. M., 22nd, he is in the following state: Drooping of the eye-lids, tears escaping on the cheek; extremities cold as ice; chest and abdomen burning hot: the latter distended, and so exceedingly painful as to make the patient scream with agony when moved. He insisted upon having cold water constantly applied, by pouring it from a vessel in which it is kept cool, but cannot support even a linen cloth. Tongue red at edges and tip, dry: pulse rapid and small; perfect consciousness, and aware that the result will be fatal. Rep. hirudines. Mustard to the inside of each thigh; stimulating frictions to the arms and legs. Evening; the friends have requested my opinion, and have begged me to allow them to do what they imagined they were able for the patient, who cannot outlive the night. He died at nine, A. M., 23rd November. Sect. cad., five hours after death. Body very stiff but warm, abdomen meteorized. In cutting through the parietes of the chest and abdomen, the skin, cellular tissue, and muscles were dry; that is, no blood escaped from their divided vessels. About ¾ of a pint of fluid were discovered in the abdominal cavity; this fluid was what has been denominated sero-purulent: that is, serum in which molecules of albumen are suspended, giving it a wheyish appearance. Some of these molecules were of considerable size, resembling shreds of false membrane. The peritoneum covering the abdominal parietes red throughout almost the whole of its extent, and covered with a thin layer of fibrine, adhering in places to its reflections over the intestines, which are inflamed in patches to a great extent. The lower portion of the ilion of a dusky red, friable, and easily lacerated. Rougeur pointillée of the cul de sac of the mucous membrane of the stomach. This organ contained a small quantity of yellowish fluid. All the other organs healthy. Blood has undergone no change; colourless clot on the right cavities of the heart." 58.

As we before remarked, we submit that the inflammation in this case was not intermittent, but only the fever.

Mr. Evans observes that the intensity of symptomatic fever might be expected to bear some proportion to its cause, but this is not the case. "In some of the most acute forms of gastritis which are found in the West Indies, the skin is cold and damp, and the pulse does not exceed 80 or 90." "Neither heat of skin nor quickness of pulse bear any proportion to the severity of the disease." This very fact is subversive of the doctrine physiologique, as far as fever is concerned. It shows that the general symptoms are *not* always dependent on the gastritis, but on other general or particular causes, as insolation or malaria. We know very well that a dose of concentrated malaria will, as it were, paralyze the powers of life, and even destroy it before any reaction takes place. These are the cases in which the gastritis is marked by the general prostration.

We shall extract one more case of "*intermittent irritation of stomach and liver cured by an emetic.*"

"A young man, aged 16, has been labouring under what he calls slight fever for the last fortnight. The paroxysms come on every morning about ten o'clock, and continue until evening; they are not preceded by rigors or even a sense of coldness; on the contrary, after a short period of malaise and slight nausea with occasional vomiting, the patient feels a heat in the feet, hands, and forehead, which soon extends itself over the whole body; he has slight thirst, and the tonge is somewhat red at the edges and tip; there is uneasiness in the epigastrium, particularly on pressure: the bowels are constipated. These symptoms are sufficiently severe to confine the patient to the sofa.—Diet, barley-water.

Our author believes that this case was one of erythema of the stomach, and thinks we owe a debt of eternal gratitude to Broussais for the following observation.

"The *sympathetic* symptoms, where the symptoms of gastric irritation are *absent*, evidently demonstrate, although indirectly, its existence."

Well done, Broussais! Well done, his credulous disciples! How are we to know that the symptoms *are sympathetic*, where there are no symptoms in the organ from whence they are supposed to proceed? Was there ever so absurd an argument as that we are to infer the *presence* of a thing by its *absence?* More than one hundred pages of the work are dedicated to the detail of cases of fevers and neuralgic affections, all of which are interesting in themselves to the young tropical practitioner, though we do not agree with the author on all points of theory. His pathological tenets will be easily gathered from the following passages.

"All these cases appear to have a common origin, and to have had for a cause, either existing or predisposing malaria. This agent, though always a predisposing cause, is not, at least in the generality of instances, the immediate or exciting one. We can most frequently trace the paroxysm to something which has disturbed the balance of the circulation or directly produced an irritation or inflammation of an organ. It is in part to the variety of exciting causes that we are to attribute the differences which exist in individual cases; but there are also other and very powerful reasons to be found for these differences, in the constitutions and idiosyncracies of the persons attacked; in the length of time that they may have resided in the country; in their professions and mode of life, &c." 202.

"The symptoms peculiar to the endemic fevers of the West Indies are those known under the name of bilious; thirst, vomiting either of bile or of porracious and mucous fluids, constant nausea; agitation; anxiety; supination; frequent sighing and moaning; tenderness of the epigastrium; frontal headache; heat of skin, and quickness of the pulse. Do not these symptoms indicate a *local origin,* and that the stomach is the suffering organ? What is there deserving the name of *essentiality* about them, more than about those which accompany an inflammation of the pulmonary parenchyma? Should they observe a type purely intermittent, can we feel more repugnance in referring them to an intermittent gastritis or gastro-hepatitis, that in referring the *son mât,* the pain, the cough, the expectoration, the crepitating râle, and the febrile symptoms, to a pneumonia? Though an inflammation of the gastro-enteric mucous membrane is not the only cause of the hot or febrile stage of an intermittent, or remittent, it is by far the most common one; and to the production of the symptoms which form the bilious fevers of the West Indies it is indispensable.

These symptoms, as we have seen, differ amongst themselves according to the circumstances under which they may have been produced. Thus the fever may be caused by exposure to the sun, and set in with a series of phænomena indicative of irritation of some part of the encephalon, and the gastric affection may not make its appearance till afterwards; or it may be caused by exposure to a current of air, and commence with a catarrh or a pneumonia. Not unfrequently it may be traced to the direct influence of malaria, in which case to a local inflammation most generally is added a diseased condition of the blood, which considerably modifies the features of the malady.

When to an intense inflammation of the gastro-intestinal canal is added a diseased state of the blood, we frequently meet with symptoms said to be *peculiar* to the yellow fever, viz. yellowness of the skin, and black vomit. In this form of the disease we have both the symptoms and cadaveric appearances met with in animals that have died from the effects of putrid matter injected into the circulation. It generally, but not always, is found in persons who have resided only a short time in the country—when exceptions are observed the cases can almost always be traced to the inhalation of malaria in a concentrated form.

The great and continued elevation of temperature within the tropics gives to all inflammations, more particularly to those of the chylopöetic viscera, a degree of violence and rapidity unknown in the temperate climates. Patients frequently die from the destruction of an organ essential to life in a space of time scarcely credible to those who have not witnessed disease in these countries.

All the diseases which we have examined may be either intermittent, remittent, or continued, and yet preserve their pathological character. It would be impossible to discover at the commencement of many of them, the type which they will assume, and in fact much will depend upon the treatment which may be pursued. Many that were at first purely intermittent become remittent or continued; and of those which set in with violent and continued inflammation of a vital organ, many may be checked or induced to put on an intermittent type by prompt and energetic treatment." 204.

We would say that the symptoms above described do not mark the "*local origin*," but the direction which the fever takes in its course. The foregoing symptoms are not the *primary* symptoms resulting from the malaria or insolation, but the *secondary* ones, which come forth when the functions or structures of particular organs, as the brain, liver, stomach, &c. are invaded and disordered. We might as well consider suppuration as the cause of inflammation, as gastritis to be the cause of fever, where general or malarious causes were demonstrably in operation. Not that we think the doctrine in question makes much difference in practice; for when any organ or part becomes affected in fever, we must attend to *that*, whether originally or secondarily disordered.

In the treatment of West India fevers, Mr. Evans adopts the practice as well as the doctrines of his master, Broussais, with some important modifications. He declaims against the mercurial treatment, and considers it as worse than no treatment at all. We conclude by strongly recommending the work to every medical man who leaves the shores of England for the West India Islands. It is full of instruction for that class of the profession, and indeed contains a great mass of materials that are interesting to the pathologist and practitioner of this country.

---

